# Ossifying Fibromyxoid Tumor with Spinal Cord Compression and Epiduritis

**DOI:** 10.5334/jbsr.1606

**Published:** 2019-02-28

**Authors:** Tessa De Wandeler, Dirk Van Gestel, Patrice Jissendi Tchofo

**Affiliations:** 1 Department of Medical Imaging, CHU Saint-Pierre, Université Libre de Bruxelles, Brussels, BE; 2 Department of Radiotherapy-oncology, Institut Jules Bordet, Université Libre de Bruxelles, Brussels, BE

**Keywords:** Myxofibrosarcoma, Scattered calcifications, Epiduritis, Spinal cord compression

## Case

**Figure 1:**
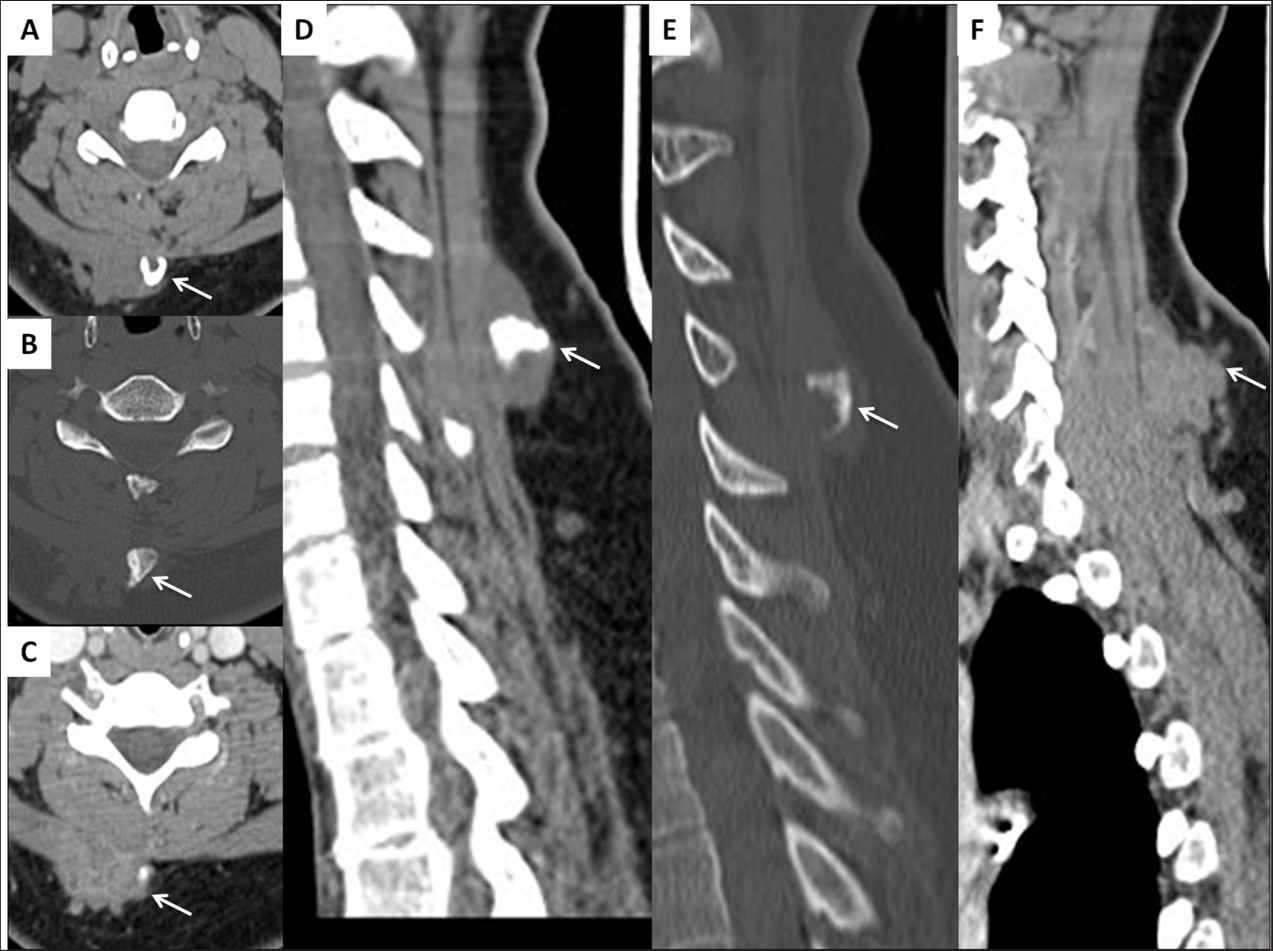


**Figure 2:**
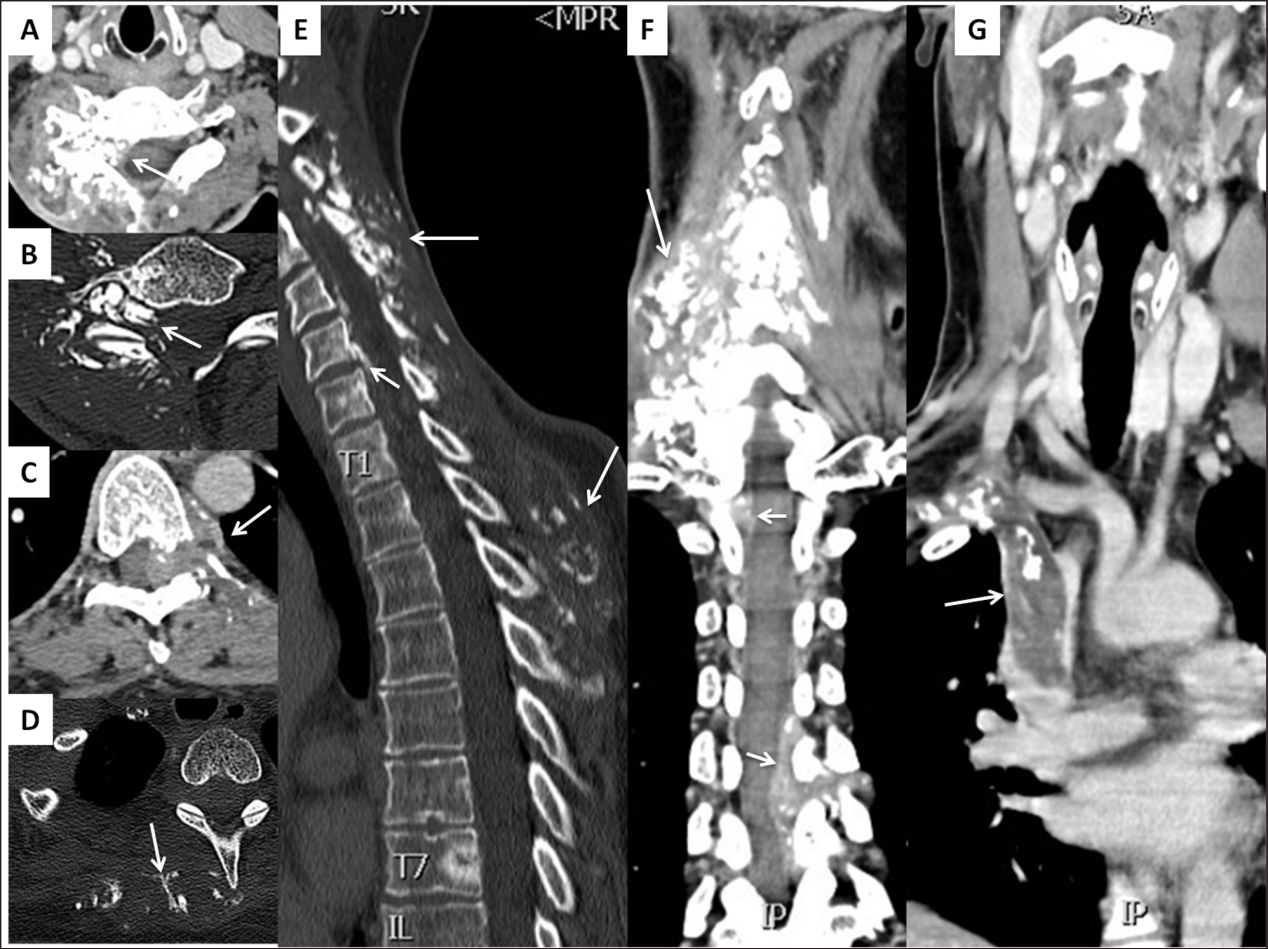


**Figure 3:**
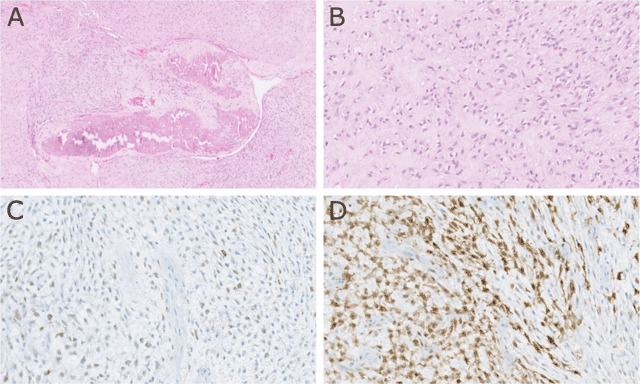


A 37-year-old female was assessed for a slowly growing right cervical mass. The mass was an asymptomatic nodule at the beginning but gave rise to neck and right upper limb pain 3–4 months later. The patient showed a swollen right upper limb with complete loss of motor function but preserved sensory function. Contrast-enhanced computed tomography (CECT) (Figure 1) revealed a soft tissue mass in tight contact with the right trapezius muscle, containing a central dysmorphic calcification. The mass is enhancing as the adjacent muscles with poor delineation with the trapezius as seen on axial (C) and sagittal (F) contrast-enhanced CT images (arrows). Shortly after, the lesion was surgically removed. Almost two years later, a control CECT (Figure 2) showed a large contrast-enhanced mass containing multiple scattered calcifications (A–E, arrows), extending into the paravertebral muscles (F, long arrow), towards the spinal canal and the foramina, with multifocal epidural invasion, compression of the spinal cord and nerve roots (F, short arrows) and elsewhere, thrombosis of the superior vena cava (G, arrow), all consistent with an extensive tumor recurrence. Unfortunately, the multiple recurrences were unsuccessfully managed by repeated surgery, radiotherapy and chemotherapy. Histopathology (Figure 3) showed typical features of ossifying fibromyxoid tumor (OFMT) with bony component.

## Comment

Ossifying fibromyxoid tumor (OFMT) is a rare soft tissue tumor of uncertain differentiation and malignancy [1]. OFMT mostly occurs in patients in their fifties with a slight male predilection. Extremities are mostly affected. OFMT shows variable histopathologic features and multiple subtypes have been described. OFMT can contain bone, osteoid and collagen elements. Radiographic features of OFMT are non-specific. It may present as a nodular soft tissue mass and in 60–70% of cases, a peripheral rim of ossification or “bone shell” can be seen. Underlying bone may be eroded or may show features of periosteal reaction. The treatment of choice is a large surgical resection of the lesion, along with a close follow-up, depending on the microscopic subtype. Recurrence is not rare, with thus a bad prognosis.
